# Successful Wide Hybridization and Introgression Breeding in a Diverse Set of Common Peppers (*Capsicum annuum*) Using Different Cultivated Ají (*C*. *baccatum*) Accessions as Donor Parents

**DOI:** 10.1371/journal.pone.0144142

**Published:** 2015-12-07

**Authors:** Juan Pablo Manzur, Ana Fita, Jaime Prohens, Adrián Rodríguez-Burruezo

**Affiliations:** 1 Instituto de Investigaciones Agropecuarias CRI La Platina, Santiago, Chile; 2 Instituto de Conservación y Mejora de la Agrodiversidad Valenciana, Universitat Politècnica de València, Camino de Vera s/n, 46022 Valencia, Spain; United States Department of Agriculture, UNITED STATES

## Abstract

*Capsicum baccatum*, commonly known as ají, has been reported as a source of variation for many different traits to improve common pepper (*C*. *annuum*), one of the most important vegetables in the world. However, strong interspecific hybridization barriers exist between them. A comparative study of two wide hybridization approaches for introgressing *C*. *baccatum* genes into *C*. *annuum* was performed: i) genetic bridge (GB) using *C*. *chinense* and *C*. *frutescens* as bridge species; and, ii) direct cross between *C*. *annuum* and *C*. *baccatum* combined with *in vitro* embryo rescue (ER). A diverse and representative collection of 18 accessions from four cultivated species of *Capsicum* was used, including *C*. *annuum* (12), *C*. *baccatum* (3), *C*. *chinense* (2), and *C*. *frutescens* (1). More than 5000 crosses were made and over 1000 embryos were rescued in the present study. *C*. *chinense* performed as a good bridge species between *C*. *annuum* and *C*. *baccatum*, with the best results being obtained with the cross combination [*C*. *baccatum* (♀) × *C*. *chinense* (♂)] (♀) × *C*. *annuum* (♂), while *C*. *frutescens* gave poor results as bridge species due to strong prezygotic and postzygotic barriers. Virus-like-syndrome or dwarfism was observed in F_1_ hybrids when both *C*. *chinense* and *C*. *frutescens* were used as female parents. Regarding the ER strategy, the best response was found in *C*. *annuum* (♀) × *C*. *baccatum* (♂) crosses. First backcrosses to *C*. *annuum* (BC_1_s) were obtained according to the crossing scheme [*C*. *annuum* (♀) × *C*. *baccatum* (♂)] (♀) × *C*. *annuum* (♂) using ER. Advantages and disadvantages of each strategy are discussed in relation to their application to breeding programmes. These results provide breeders with useful practical information for the regular utilization of the C. *baccatum* gene pool in *C*. *annuum* breeding.

## Introduction

Crop relatives have been used for decades for breeding, in particular to transfer genes of resistance or tolerance to pests, diseases or abiotic stress to the cultivated species [1, 2]. Introgression breeding has been extensively used in the genetic improvement of some of the most important *Solanaceae* crops, like potato (*Solanum tuberosum* L.) or tomato (*Solanum lycopersicum* L.). Thus, up to twelve traits have been introgressed in potato from related species like *S*. *demissum*, *S*. *stoloniferum*, *S*. *chacoense*, *S*. *acaule*, *S*. *vernei* or *S*. *spegazzinii* [3] and many more have been transferred to tomato from their wild relatives like *S*. *peruvianum*, *S*. *cheesmanii*, *S*. *pennellii* or *S*. *chilense* [4]. However, breeding programmes in the economically important common *Capsicum* peppers (*Capsicum annuum* L.) have made little use of related species for breeding as reviewed by Mongkolporn and Taylor [5]. This limitation has been mainly due to the presence of different pre-zygotic barriers which avoid fertilization (e.g. pollen-pistil incompatibilities) and/or post-zygotic barriers, which prevent the achievement of fertile hybrids, e.g. embryo/endosperm abortion, hybrid weakness or sterility [6, 7].

In this sense, *C*. *annuum* is related to about other 30 *Capsicum* species, of which four are also cultivated, *C*. *baccatum* L., *C*. *chinense* Jacq., *C*. *frutescens* L. and *C*. *pubescens* R. & P. [8]. By one hand, *C*. *chinense* and *C*. *frutescens* cultivars have economic importance in America, Africa, and Asia and both are phylogenetically close to *C*. *annuum*. In fact, these species make up the *annuum-chinense-frutescens* complex (or *annuum* complex), characterized by white flowers and yellow seeds [9]. On the other hand, *C*. *pubescens* and *C*. *baccatum* represent separate taxons from the *annuum* complex and, although they have been widely grown in the Andean region and Brazil for millennia, they are very rare outside this area nowadays [9, 10]. The former, mainly known as rocoto (with purple flowers and black rough seeds), is the least economically important [9]. Due to prezygotic barriers which prevent the growth of the pollen tube through the style, and possible postzygotic barriers, it does not cross with any of the other four species [9, 11]. The latter, commonly known as ají (with white flowers and yellow spots), has showed an extremely low/nil cross compatibility with *C*. *annuum* [6], although it has been reported as source of variation for a range of traits with potential interest for the genetic improvement of this species. These include resistances to several diseases such as: anthracnose (*Colletotrichum spp*.), powdery mildew (*Leveillula taurica*), *Rhizoctonia* root rot (*Rizhoctonia solani*), *Verticillium* wilt (*Verticilium dahliae*) and bacterial wilt (*Ralstonia solanacearum* syn. *Pseudomonas solanacearum*), viruses like PYMV and TSWV or even new flavours [12–21]. However, successful wide hybridization attempts to introgress these traits in *C*. *annuum* have been scarce [12, 22].

Postzygotic barriers have been suggested as the main cause of cross compatibility problems between both species, specifically embryo/endosperm abortion and hybrid sterility [23, 24]. In most plant species, the first barrier is caused by abnormal cell division of the zygote or slow endosperm development, which causes an incompatibility with embryo growth [25], while the second is due to a range of factors such as diverged genes, karyotypic changes, gene transposition or gene loss, sequence divergence or dosage imbalance, among others [26].

An alternative to overcome these barriers, known as genetic bridge, is based on the use of species (bridges) phylogenetically close to the two species affected by crossability barriers. The bridge species is used to obtain hybrids with one of the target species, and subsequently these hybrids are crossed to the other target species [27]. Thus, *C*. *chinense* and *C*. *frutescens* might play this role for wide hybridization between *C*. *annuum* and *C*. *baccatum* as previously suggested by Pickersgill [28].

Another strategy for wide hybridization between *C*. *annuum* and *C*. *baccatum* is the *in vitro* rescue of immature interspecific embryos or embryo rescue before abortion occurs [27]. This approach is technically more complex as it requires embryo excision and *in vitro* culture. Also, the stage at which embryo abortion occurs after hybridization may depend on the specific genotypes involved in the cross. Thus, for example within *Solanaceae*, while some authors could rescue interspecific embryos at the latest immature stages [29], there are also examples on which embryos had to be rescued at the earliest stages [30, 31]. However, the earlier the stage at which embryo rescue is done, the more difficult is the procedure and the lower the efficiency [32].

Furthermore, even though hybrid materials could be achieved, hybrid sterility must be also considered as an important postzygotic barrier. Full sterility or different degrees of fertility of the interspecific hybrids may vary depending on the parent genotypes. In extreme cases, when sterility is complete due to the lack of chromosome pairing during meiosis, fertility may be restored by poliploidization, enabling pairing of homologous chromosomes in the allopolyploid hybrid [33].

Unfortunately, studies on wide hybridization between *C*. *baccatum* and *C*. *annuum* and the overcoming of their compatibility barriers are very scarce [29, 34], especially regarding the range of diversity encompassed in materials used for crosses. Consequently, there is a lack of detailed practical information about the breeding process and levels of sexual compatibility among species, which might also depend on the genotypes involved or the direction of the crosses, among other factors.

Therefore, the development of approaches which allow overcoming these barriers, assessing all the difficulties which can appear during their application, will provide breeders with useful tools, practical information and a comprehensive perspective for the introgression of genes of interest from *C*. *baccatum* to *C*. *annuum*. Moreover, the use of a wide genetic diversity will contribute to offer a more complete view of these barriers.

The aim of this work was to compare comprehensively two approaches for the achievement of wide hybridization between *C*. *annuum* and *C*. *baccatum*: i) genetic bridge using *C*. *chinense* and *C*. *frutescens* as bridge species; and, ii) direct hybridization between *C*. *annuum* and *C*. *baccatum* in combination with embryo rescue. In both strategies full diallel interspecific crosses were planned and a range of genetically different genotypes were used. The effects of the direction of the crosses, cross compatibility at different levels among the species involved and hybrid viability and fertility are also discussed.

## Material and Methods

### Plant material and growing conditions

A total of 18 accessions from four cultivated species of *Capsicum* were utilized in the present study: *C*. *annuum* (12 accessions), *C*. *baccatum* (3 accessions), and the bridge species *C*. *chinense* (2 accessions) and *C*. *frutescens* (1 accession). This collection encompassed a comprehensive range of geographical origins and fruit morphological traits (Table 1). New materials (i.e. interspecific hybrids and backcrosses) were included progressively in the experiments as they were obtained during the wide hybridization programme. Plants from parent accessions, and subsequent hybrids and backcrosses were transplanted at the four-leaf stage (about 50–60 days after sowing) to glasshouses at the Universitat Politècnica de València (UPV, Valencia, Spain) along three years (2010, 2011 and 2012). Plants were grown in 10 L pots (coconut coir as substrate), under the spring-summer growing cycle as it provides the most favourable conditions for the development and fruit set of *Capsicum* in the Mediterranean region [8]. Natural illumination was used for this experiment and temperature control systems (heating and cooling) were activated when temperatures dropped below 18°C (night) or rose above 25°C (day). Plants were pruned to four stems and trained with vertical strings. They were drip irrigated every 8 h for 3 min (4 L/h). Fertilizer was applied with the irrigation water, at a rate of 1 g/L of a commercial 15N-2.2P-24.9K water soluble fertilizer (BASF, Barcelona, Spain).

**Table 1:** Origin and fruit traits of the accessions utilized in the present experiment.

Species	Accession	Code	Origin	Color	Weight (g)	Length (cm)	Width (cm)	Pollen viability (%±SD)
*C*. *annuum*	Arnoia	Arn	Centro Investigaciones Agrarias de Magebondo (Galicia, Spain)	Red	50–90	7–11	5–7	82±3
*C*. *annuum*	Bierzo	Bie	Cons. Reg. IGP Pimiento Asado Bierzo, Ponferrada (León, Spain)	Red	4–7	6–7	4–8	85±5
*C*. *annuum*	California Wr. red	CWr	Breeding line, UPV-COMAV	Pale red	90–150	8–11	6–10	91±4
*C*. *annuum*	California Wr. yellow	CWy	Breeding line, UPV-COMAV	Yellow	108–176	8–10	7–11	94±4
*C*. *annuum*	Guindilla	Gui	Guindilla de Ibarra, Ibarra, Spain	Deep red	8–14	8–15	1–2	98±1
*C*. *annuum*	Numex	Num	New Mexico State University, USA	Red	30–70	12–15	4–6	83±3
*C*. *annuum*	Pasilla Bajío	Pas	Mexico, Southern USA	Brown	20–30	15–20	2–3	89±4
*C*. *annuum*	Pimiento de Bola	Bola	Cons. Reg. DOP Pimentón Murcia, Murcia, Spain	Red	10–14	3–5	4–6	94±2
*C*. *annuum*	Pimiento del Piquillo	Piq	Cons. Reg. IGP Piquillo Lodosa, Navarra, Spain	Red	20–33	7–9	4–6	85±3
*C*. *annuum*	PBC534	P534	Asian Vegetables Research and Development Center (AVRDC)	Red	8–15	10–15	1–2	86±4
*C*. *annuum*	PBC716	P716	Asian Vegetables Research and Development Center (AVRDC)	Red	2–5	4–8	>0.5	93±4
*C*. *annuum*	Serrano	Ser	Mexico	Deep red	3–5	3–5	1–2	89±5
*C*. *chinense*	Ají Panca	AjíP	Ecuador	Brown	10–14	7–10	2–3	81±3
*C*. *chinense*	PI-152225	PI15	USDA	Deep red	4–6	4–5	1–2	84±2
*C*. *frutescens*	Bol 144	B144	Bolivia	Pale red	>1	1–2	>0.5	92±4
*C*. *baccatum*	Ají Rojo	AjíR	Bolivia	Red	5–8	5–8	2–3	86±3
*C*. *baccatum*	Ají Amarillo	AjíA	Bolivia	Yellow	3–4	4–6	1–2	91±2
*C*. *baccatum*	Brazilian pumpkin	BrP	Brazil	Red	2–3	2–3	2–3	84±3

### Hybridization technique

Previously to hybridization, female flowers were emasculated and pollen was extracted from male flowers and released carefully on the stigma. To prevent uncontrolled pollination, female flowers were covered with Scotch tape after hybridization. Each cross was tagged with the genotypes involved in the hybridization and the date at which it was performed. Overall, more than 5000 hybridizations were performed in this study.

### Genetic bridge approach

**Fig 1:**
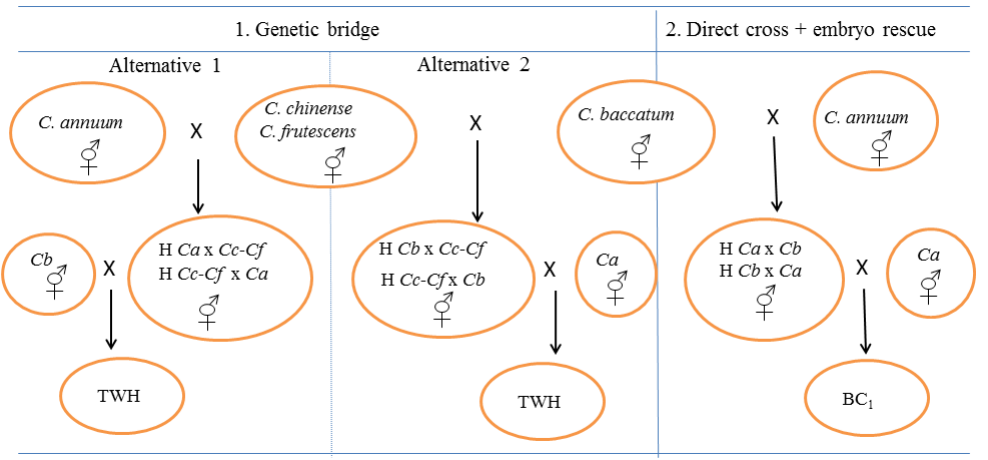
Diagram of genetic bridge (GB) and embryo rescue (ER) planned approaches to overcome interspecific barriers between *C*. *annuum* and *C*. *baccatum*. Vertical arrows indicate the hybrid obtained, H = Hybrid, Ca = *C*. *annuum*, Cb = *C*. *baccatum*, Cc = *C*. *chinense*, Cf = *C*. *frutescens*, BC_1_ = First backcross, TWH = Three way hybrid.

Within the genetic bridge strategy (GB), all possible combinations of full (reciprocal) diallel crosses between the 12 *C*. *annuum* and the three *C*. *baccatum* accessions on one side and the three accessions of the bridge species *C*. *chinense* (2 accessions) and *C*. *frutescens* (1 accession) on the other were tested (i.e. a total of 45 hybrid combinations and their reciprocals). F_1_ hybrid materials obtained were further used for completing the bridge cross (i.e., crossing to *C*. *annuum* if *C*. *baccatum* had been used to obtain the F_1_, and *viceversa*). Between 10 and 30 hybridizations were done for each cross combination and direction of the cross. Two strategies were performed to achieve the bridge cross (Fig 1): i) alternative 1, which firstly consisted in crossing *C*. *annuum* with the bridge species and, later the obtained hybrids were crossed with *C*. *baccatum*; and, ii) alternative 2, which firstly consisted in crossing *C*. *baccatum* with the bridge species and, later the obtained hybrids with *C*. *annuum*. As a result, a total of over 2800 hybridizations were done in this GB approach. Fully ripe fruits from each successful cross were harvested and their seeds were extracted and dried. After that, seeds were sown in seedling trays (7×12 cells, 480×300×55 mm) containing cultivation substrate (Humin-substrat N3, Klasmann-Deilmann, Germany). Once plantlets reached the four-leaf stage, they were transferred to glasshouses to perform the second cross (hybrid × parental cross) in order to obtain three-way hybrids (Fig 1).

### Embryo rescue

Within the embryo rescue strategy (ER), a minimum of 10 hybridizations (and up to 47 in some cross combinations) between the 12 *C*. *annuum* accessions on one side and the three *C*. *baccatum* accessions on the other were performed using a full diallel scheme (i.e., 36 hybrid combinations) with reciprocals. Also several BC_1_ crosses towards *C*. *annuum* were later tried on the basis of available hybrids. As a result, a total of 2500 hybridizations were done in the present ER experiment. About 25–30 days after pollination immature fruits were harvested to perform the embryo rescue following the protocol and culture medium optimized by us in previous studies [35, 36]. The medium included: agar (7 g/L), indole-3-acetic acid (IAA, 0.01 mg/L), gibberellic acid (GA_3_, 0.01 mg/L), zeatin (0.01 mg/L), sucrose (40 g/L), and MS (2.2 g/L), at pH 5.7. The basal medium (agar, sugars and MS salts) was sterilized at 121°C for 20 min. Hormones were sterilized by microfiltration (0.20 μm Minisart® filters) and were added to the warm (35–40°C) autoclaved medium before solidifying. Embryos were excised carefully and immediately cultured in 90×15 mm Petri dishes, which were sealed with Parafilm® and incubated in a growth chamber (25±10°C; 70% HR; full darkness the first 5 days and a 16h/8h (light/dark) photoperiod since day 6). About 1000 immature seeds were dissected for the ER approach. After 30 days of incubation, seedlings which showed a clear development of root and shoot were transferred to seedling trays and were covered with perforated plastic glasses to prevent dehydration. After one week, plastic glasses were removed. Once F_1_ plantlets reached the four-leaf stage, they were transferred to 10 L pots at glasshouses for evaluation and to obtain subsequent BC_1_ generations (Fig 1).

### Cross compatibility evaluation and confirmation of hybridity

In both strategies the compatibility of each cross was evaluated at four levels: i) number of fruits set per number of hybridizations performed, ii) percentage of germinated seeds (or rescued embryos in the case of ER) 1 month after sowing, iii) plant hybrid appearance, and iv) pollen viability (n = 5 mesurements), estimated according to Rodríguez-Riaño and Dafni [37] with 1% (w/v) of thiazolyl blue tetrazolium bromide (MTT) dye (M-2128, Sigma-Aldrich). To confirm the hybrid nature of new materials, we used a set of six polymorphic SSRs (Table 2). These SSRs markers were scored according to the method described by Minamiyama et al. [38].

**Table 2:** SSRs utilized to confirm the hybridity of individuals obtained after interspecific hybridization [37].

SSRs	Primer forward	Primer reverse	Size (pb)	Linkage group
CAMS-679	TTTGCATGTTTTACCCATTCC	ATGTGAAACACATAGGTAGCACTGA	200	1
CAMS-460	CCTTTCACTTCAGCCCACAT	ACCATCCGCTAAGACGAGAA	230	7
CAMS-405	TTCTTGGGTCCCACACTTTC	AGGTTGAAAGGAGGGCAATA	260	11
CAMS-644	CGCATGAAGCAAATGTACCA	ACCTGCAGTTTGTTGTTGGA	200	4
CAMS-806	TGTCACAAGTGTCAAGGTAGGAG	CCCCAAAAATTTTCCCTCAT	140	10
CAMS-398	ATGGTCCATGGTCAGCAGAT	GGGCAGAACAGTGGATGATT	180	7

## Results and Discussion

Both approaches (GB and ER) enabled to successfully achieve the wide hybridization between *C*. *annuum* and *C*. *baccatum*, including obtaining F_1_ hybrids, three-way-hybrids, and even backcross generations within the ER strategy. On the basis of the SSRs analysis, all new materials were confirmed as hybrids of their corresponding parents and, therefore, no uncontrolled pollination was present in the experiment. This is an important step for *C*. *annuum* breeding using *C*. *baccatum* as donor species for traits present in the latter. In the case of the ER approach first backcross (BC_1_) generations towards *C*. *annuum* were obtained; and, in the case of the GB approach three-way hybrids using *C*. *chinense* as bridge species from both cross alternatives (i.e. starting with crosses between *C*. *annuum* and *C*. *chinense* and between *C*. *baccatum* and *C*. *chinense*) were achieved. However, a range of results, efficiency rates and crossability barriers was found in both strategies depending on the direction and the genotypes involved in the crosses. Consequently, all these factors must be considered to plan breeding programs which include interspecific crosses between these four species.

### Genetic bridge approach

#### *C*. *chinense* as genetic bridge species

**Fig 2:**
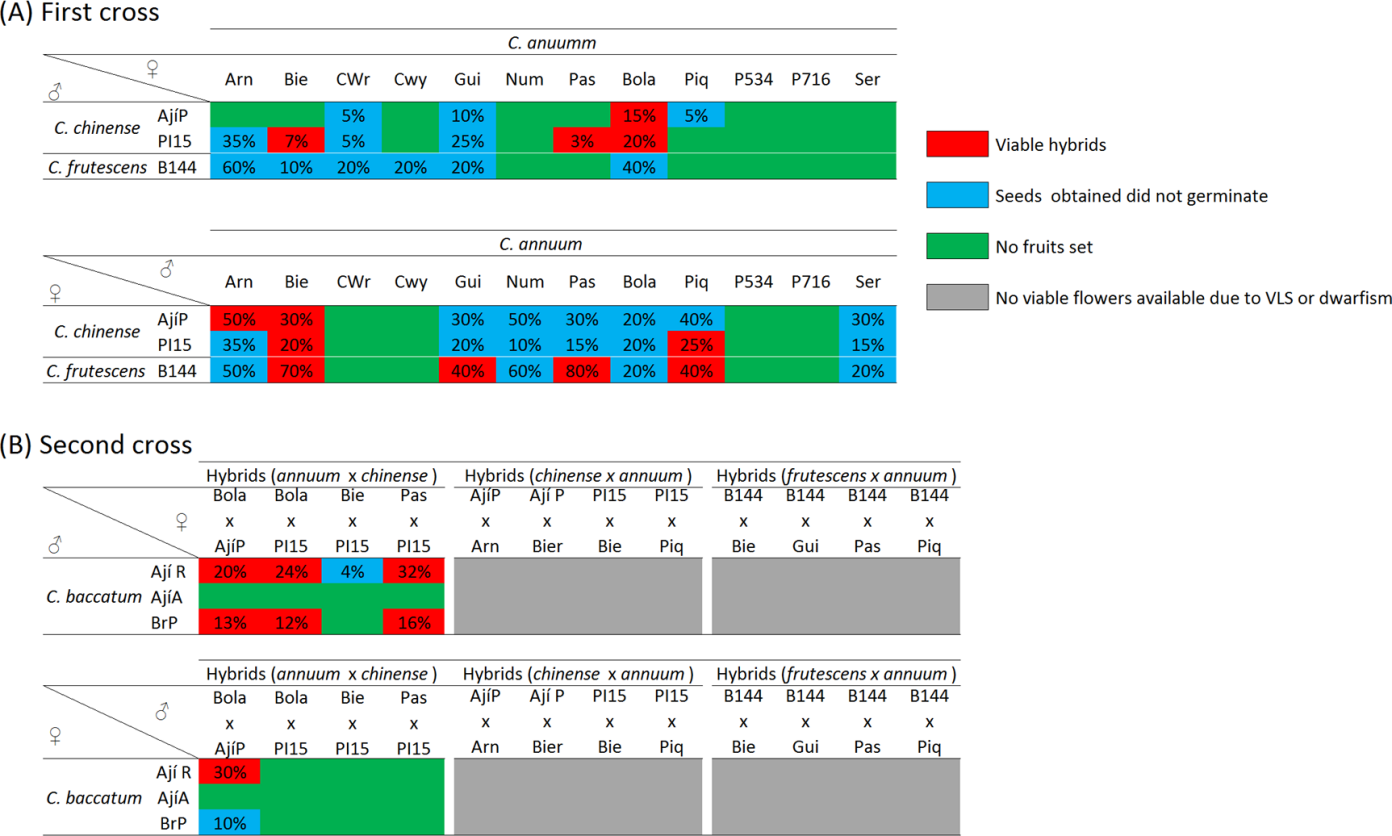
Cross diagram showing the results of the different crosses performed within the genetic bridge approach starting with crosses between *C*. *annuum* and the bridge species. (A) First cross table indicates the crossability degree of *C*. *annuum* with *C*. *chinense* or *C*. *frutescens*, (B) Second cross table indicates the crossability degree of hybrids obtained in A with *C*. *baccatum*. Numbers over the cells indicate percentage of fruit set over an average of 25 artificial pollinations. Green indicates non-viable crosses, where no fruits were obtained (0% of fruits set, not indicated as number); blue indicates fruit set but non-viable seeds; red indicates fertile hybrids with normal development; and grey indicates hybrid inviable plants.

Despite a high crossability between the species of the *annuum* complex has been widely assumed [6, 8, 9], many crosses of *C*. *annuum* accessions with both *C*. *chinense* or *C*. *frutescens* were unsuccessful (Fig 2). Thus, in the *C*. *annuum* (♀) × *C*. *chinense* (♂) cross scheme, fruit set occurred in 10 of the 24 possible combinations and only three *C*. *annuum* accessions (California Wr. red, Bola and Guindilla) were able to set fruit with any of the *C*. *chinense* accessions used (Fig 2). Furthermore, only the seeds of four combinations germinated and developed in normal hybrids, which also showed pollen viability estimates comprised between 17–31% (Table 3), considerably lower than those from their parent accessions, always higher than 80% (Table 1). *Capsicum annuum* accession Bola was the only one which produced hybrid plants with both *C*. *chinense* accessions (Table 3). Moreover, one of these (Bola × P15) was the hybrid with the highest pollen viability (31%). Therefore, this accession appears as suitable germplasm for the GB strategy, as well as for the transfer of genes from *C*. *chinense* to commercial peppers. Although Zijlstra et al. [11] discarded prezygotic barriers between species belonging to the *annuum* complex, the low or even nil fruit set rates obtained in our experiment suggest strong prezygotic incompatibility between *C*. *annuum* and *C*. *chinense*. Even more, as observed in some cases by Inai et al. [39], our low germination rates could be due to embryo abortion, which also suggest postzygotic barriers.

**Table 3:** Descriptive results of the genetic bridge (GB) strategy using the alternative of obtaining hybrids between *C*. *annuum* (Ca) and *C*. *chinense* (Cch) for being subsequently crossed with *C*. *baccatum* to obtain three-way hybrids. Cross combinations that did not set fruit or set fruit but seeds did not germinate are not included in the table.

Pistillate (♀) accessions	×	Staminate (♂) accessions	No. crosses	No. set fruit (fruit/crosses %)		Germination (%)	Hybrid appearance	Pollen viability (% ± SD)
*C*. *annuum*	×	*C*. *chinense*						
Bie	×	P15	30	2	(7%)	10/15 (67%)	Normal	21 ± 4
Bola	×	AjíP	20	3	(15%)	3/14 (21%)	Normal	17 ± 5
Bola	×	P15	20	4	(20%)	4/15 (27%)	Normal	31 ± 6
Pas	×	P15	30	1	(3%)	4/14 (29%)	Normal	18 ± 8
*C*. *chinense*	×	*C*. *annuum*						
AjíP	×	Arn	20	10	(50%)	9/14 (64%)	VLS^1^	-
AjíP	×	Bie	20	6	(30%)	6/15 (40%)	VLS	-
PI15	×	Bie	20	4	(20%)	1/15 (7%)	VLS	-
PI15	×	Piq	20	5	(25%)	6/15 (40%)	VLS	-
(*Ca* × *Cch*)	×	*C*. *baccatum*						
Bola × AjíP	×	AjíR	15	3	(20%)	6/14 (43%)	Normal	83 ± 6
Bola × AjíP	×	BrP	15	2	(13%)	8/14 (57%)	Normal	28 ± 5
Bola × PI15	×	AjíR	25	6	(24%)	1/14 (7%)	Normal	33 ± 6
Bola × PI15	×	BrP	25	3	(12%)	7/14 (50%)	Normal	12 ± 4
Pas × PI15	×	AjíR	25	8	(32%)	11/14 (79%)	Normal	15 ± 7
Pas × PI15	×	BrP	25	4	(16%)	3/14 (21%)	Normal	4 ± 3
*C*. *baccatum*	×	(*Ca* × *Cch*)						
AjíR	×	Bola × AjíP	10	3	(30%)	1/5 (20%)	Normal	95 ± 2

^1^ VLS = virus-like syndrome.

**Fig 3:**
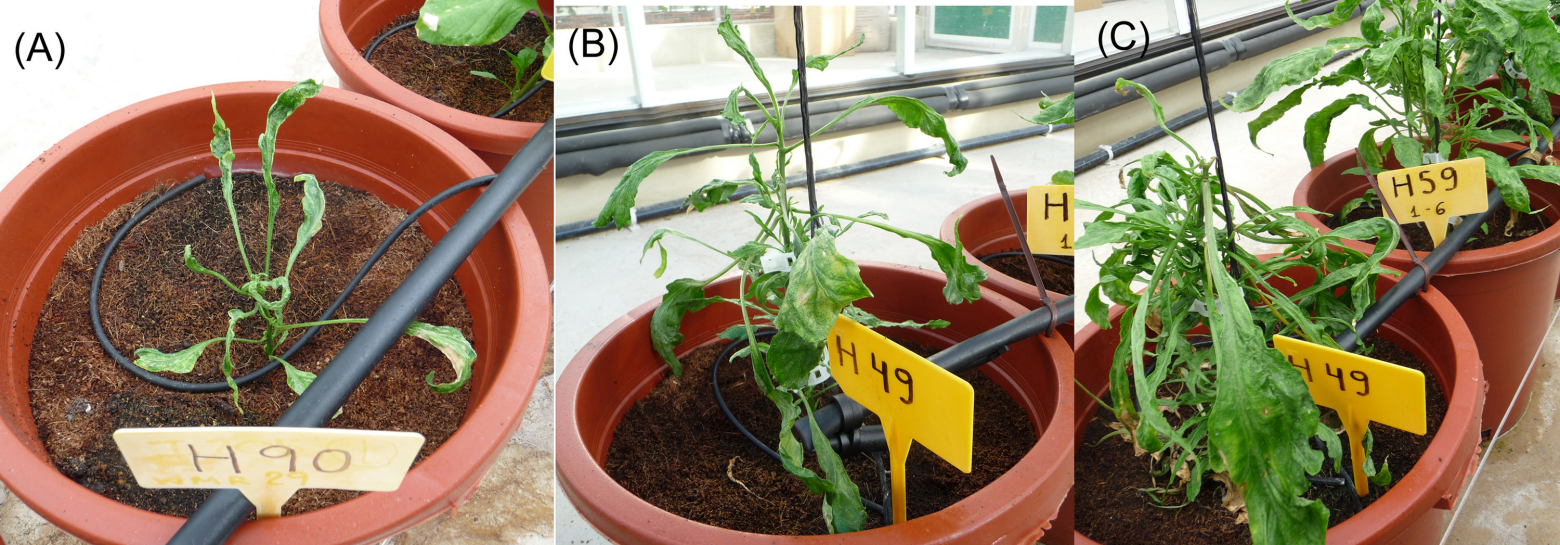
*C*. *chinense* (♀) × *C*. *annuum* (♂) hybrids showing virus-like syndrome (VLS). (A) at 50 days after sowing, (B) 100 days after sowing, and (C) 150 days after sowing. Stunted growth, filiform leaves and short internodes can be observed.

The reciprocal cross *C*. *chinense* (♀) × *C*. *annuum* (♂) enabled a higher number of successful combinations in terms of fruit set, with 16 out of the 24 possible combinations (Fig 2). However, only hybrid seeds from four combinations germinated (Table 3) and, moreover, hybrids showed stunted growth, filiform leaves, short internodes and did not enter into the reproductive phase (Fig 3). These symptoms were not observed in other plants grown in the same trial and all samples were negative to ELISA tests for Tobamoviruses or tomato spotted wilt virus (TSWV). Thus, the most likely reason for this abnormal development was the virus-like syndrome (VLS), which might be due to the interaction between cytoplasm and nuclear genes of *C*. *chinense* and *C*. *annuum* respectively [6, 39]. Therefore, the results suggest that *C*. *chinense* should always be utilised as male parent to achieve viable hybrids with *C*. *annuum*.

The four viable *C*. *annuum* (♀) × *C*. *chinense* (♂) hybrids available were then utilized to complete the bridge cross with *C*. *baccatum*. Crosses involving *C*. *baccatum* Aji amarillo did not set any fruit (Fig 2), suggesting a low cross compatibility of this accession. Then, regarding the rest of combinations, the best results were obtained using hybrids as female parents, with seven out of the eight possible combinations setting fruit, and viable seeds being obtained from six combinations (Fig 2 and Table 3). All these materials showed a normal appearance, although a wide range of pollen viability was observed, which was comprised between 4% and 83% in (Pas × PI15) (♀) × BrP (♀) and (Bola × AjíP) (♀) × AjíR (♂) respectively (Table 3). On the contrary, when *C*. *annuum* (♀) × *C*. *chinense* (♂) hybrids were utilized as pollen donors, fruit set and successful hybrid plants were only achieved in AjíR (♀) × (Bola × AjíP) (♂) (Fig 2). Surprisingly, this hybrid showed the highest pollen viability (95%) among all hybrid materials of the GB approach involving *C*. *chinense* as bridge species, which suggests that the involved genotypes Bola (*C*. *annuum*), AjíP (*C*. *chinense*), and AjíR (*C*. *baccatum*) have a high compatibility. Consequently, given the number of successful combinations (6/8), the following scheme [*C*. *annuum* (♀) × *C*. *chinense* (♂)] (♀) × *C*. *baccatum* (♂) is recommended when using the GB approach.

**Fig 4:**
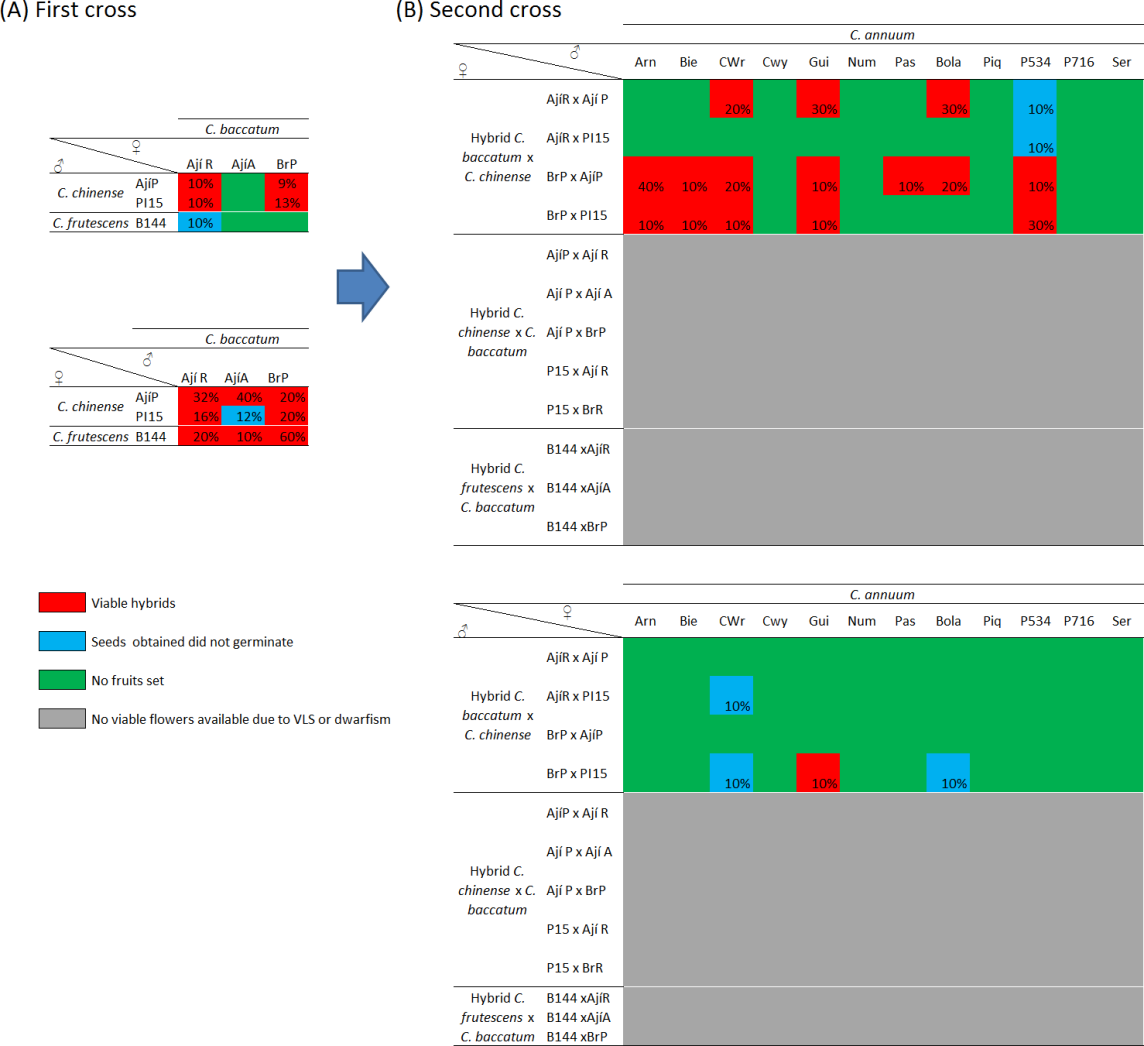
Cross diagram showing the results of the different crosses performed within the genetic bridge approach starting with crosses between *C*. *baccatum* and the bridge species. (A) First cross tables indicate the crossability degree of *C*. *baccatum* with *C*. *chinense* and *C*. *frutescens*, (B) Second cross tables indicate the crossability degree of the hybrids obtained with *C*. *annuum*. Numbers over the cells indicate percentage of fruit set over an average of 25 artificial pollinations. Green indicates non-viable crosses, where no fruits were obtained (0% of fruits set, not indicated as number); blue indicates fruit set but non-viable seeds; red indicates fertile hybrids with normal development; and grey indicates hybrid inviable plants.

Regarding the bridge cross strategy that involved *C*. *baccatum* to obtain the first set of interspecific hybrids, it was possible to achieve normal *C*. *baccatum* (♀) × *C*. *chinense* (♂) hybrids from all combinations, with the only exception of those combinations involving the Ají Amarillo accession (*C*. *baccatum*), which did not to set any fruit (Fig 4) and confirmed its low aptitude for interspecific crosses. Despite these hybrids had low pollen viability (19–33%) (Table 4), similarly to *C*. *annuum* (♀) × *C*. *chinense* (♂) materials, it was possible to obtain subsequent crosses with *C*. *annuum*.

**Table 4:** Descriptive results of the genetic bridge (GB) strategy using the alternative of obtaining hybrids between *C*. *baccatum* (Cb) and *C*. *chinense* (Cch) for being subsequently crossed with *C*. *annuum* to obtain three-way hybrids. Cross combinations that did not set fruit or set fruit but seeds did not germinate are not included in the table.

Pistillate (♀) accessions	×	Staminate (♂) accessions	No. crosses	No. set fruit (fruit/crosses %)		Germination (%)	Hybrid appearance	Pollen viability (% ± SD)
*C*. *baccatum*	×	*C*. *chinense*						
AjíR	×	AjíP	40	4	(10%)	14/14 (100%)	Normal	24 ± 9
AjíR	×	PI15	40	4	(10%)	14/14 (100%)	Normal	30 ± 12
BrP	×	AjíP	35	3	(9%)	10/10 (100%)	Normal	33 ± 10
BrP	×	PI15	40	5	(13%)	2/15 (13%)	Normal	19 ± 3
*C*. *chinense*	×	*C*. *baccatum*						
AjíP	×	AjíR	25	8	(32%)	7/15 (47%)	VLS^1^	-
AjíP		AjíA	25	10	(40%)	10/15 (67%)	VLS	-
AjíP		BrP	10	2	(20%)	1/15 (7%)	VLS	-
PI15	×	AjíR	25	4	(16%)	6/15 (40%)	VLS	-
PI15		BrP	10	2	(20%)	1/15 (7%)	VLS	-
(*Cb* × *Cch*)	×	*C*. *annuum*						
AjíR × AjíP	×	CWr	10	2	(20%)	3/6 (50%)	Normal	34 ± 12
AjíR × AjíP	×	Bola	10	3	(30%)	1/6 (17%)	Normal	10 ± 5
AjíR × AjíP	×	Gui	10	3	(30%)	2/3 (67%)	Normal	84 ± 6
BrP × AjíP	×	CWr	10	2	(20%)	10/29 (35%)	Normal	83 ± 10
BrP × AjíP	×	Arn	10	4	(40%)	12/45 (27%)	Normal	94 ± 1
BrP × AjíP	×	Bie	10	1	(10%)	2/15 (13%)	Normal	83 ± 5
BrP × AjíP	×	Bola	10	2	(20%)	4/11 (36%)	Normal	55 ± 12
BrP × AjíP	×	Gui	10	1	(10%)	3/18 (17%)	Normal	55 ± 8
BrP × AjíP	×	P534	10	1	(10%)	1/4 (25%)	Normal	11 ± 3
BrP × AjíP	×	Pas	10	1	(10%)	1/8 (13%)	Ms^2^	0
BrP × PI15	×	CWr	10	1	(10%)	5/7 (71%)	Normal	87 ± 6
BrP × PI15	×	Arn	10	1	(10%)	3/4 (75%)	Normal	58 ± 9
BrP × PI15	×	Bie	10	1	(10%)	3/3 (100%)	Normal	68 ± 10
BrP × PI15	×	Gui	10	1	(10%)	7/11 (64%)	Normal	10 ± 2
BrP × PI15	×	P534	10	3	(30%)	5/22 (23%)	Ms	0
*C*. *annuum*	×	(*Cb* × *Cch*)						
Gui	×	BrP × PI15	10	1	(10%)	1/10 (10%)	Normal	3 ± 2

^1^ VLS = virus-like syndrome

^2^Ms = male sterility.

By contrast, within the reciprocal crosses *C*. *chinense* (♀) × *C*. *baccatum* (♂), fruit set and viable seeds were observed in all possible combinations (Fig 4). Unfortunately, as observed in *C*. *chinense* (♀) × *C*. *annuum* (♂) plants, all these hybrids also showed VLS, indicating the presence of detrimental genes in *C*. *chinense* cytoplasm that may also induces detrimental effects in hybrids with *C*. *baccatum* (Table 4). This is consistent with other works related to interspecific hybridization in pepper [6, 7, 39, 40]. Therefore, as suggested for crosses with *C*. *annuum*, *C*. *chinense* should be also utilized as pollen donor in crosses with *C*. *baccatum* to prevent detrimental effects in the hybrid offspring.

Finally, the use of *C*. *baccatum* (♀) × *C*. *chinense* (♂) hybrids as male parents for finishing the GB approach only succeed in 1 out of 48 possible combinations (and in one single plant), which also showed a 3% pollen viability (Fig 4, Table 4). Such findings are in agreement with those observed in the *C*. *baccatum* (♀) × [*C*. *annuum* (♀) × *C*. *chinense* (♂)](♂) scheme, confirming that due to the frequently low pollen viability of both interspecific hybrids, *C*. *annuum* (♀) × *C*. *chinense* (♂) and *C*. *baccatum* (♀) × *C*. *chinense* (♂), they should not be used as male parents. On the contrary, the best results were achieved utilizing *C*. *baccatum* (♀) × *C*. *chinense* (♂) hybrids as pistillate parent, which allowed finishing the GB in 15 out of the 48 possible combinations (Fig 4 and Table 4). In addition, many of these hybrids showed high levels of pollen viability (>50%). Therefore, this alternative is more efficient for the GB when starting the cross scheme crossing *C*. *baccatum* with *C*. *chinense*.

As a whole, the comparison between the different alternatives for GB showed that the use of [*C*. *annuum* (♀) × *C*. *chinense* (♂)] (♀) × *C*. *baccatum* (♂) crossing schemes allowed obtaining plants with a range of pollen viability (4–83%) in six cross combinations. The use of the [*C*. *baccatum* (♀) × *C*. *chinense* (♂)] (♀) × *C*. *annuum* (♂) scheme provided 13 fertile combinations with up to 94% pollen viability. Therefore, according to these findings, we recommend to utilize the latter alternative to introgress genes from *C*. *baccatum* to *C*. *annuum*. Moreover, this strategy provides 50% *C*. *annuum* genome, while three-way hybrids from the other scheme will only carry 25% of *C*. *annuum* genome. Nevertheless, it should be also considered that this strategy, depending on the character to introgress, may require checking the resulting phenotypes at the first step from crosses between *C*. *baccatum* and *C*. *chinense* before performing the second cross involved in the bridge cross. In fact, Yoon and Park (2005) [12] utilized the other alternative, despite they also found low pollen viability.

#### *C*. *frutescens* as genetic bridge species

In comparison to *C*. *chinense*, *C*. *frutescens* showed more difficulties to be used as a bridge species. Thus, although *C*. *frutescens* is phylogenetically close to *C*. *annuum* and it is generally supposed that hybridization between both species can be done easily [9], results suggest that such compatibility is not so obvious. In this way, when *C*. *frutescens* was utilised as pollen donor with *C*. *annuum*, hybrid seeds did not germinate (Fig 2), which is consistent with other authors who have reported some cases of embryo abortion and even after using embryo rescue they obtained partially sterile hybrids [41].

**Fig 5:**
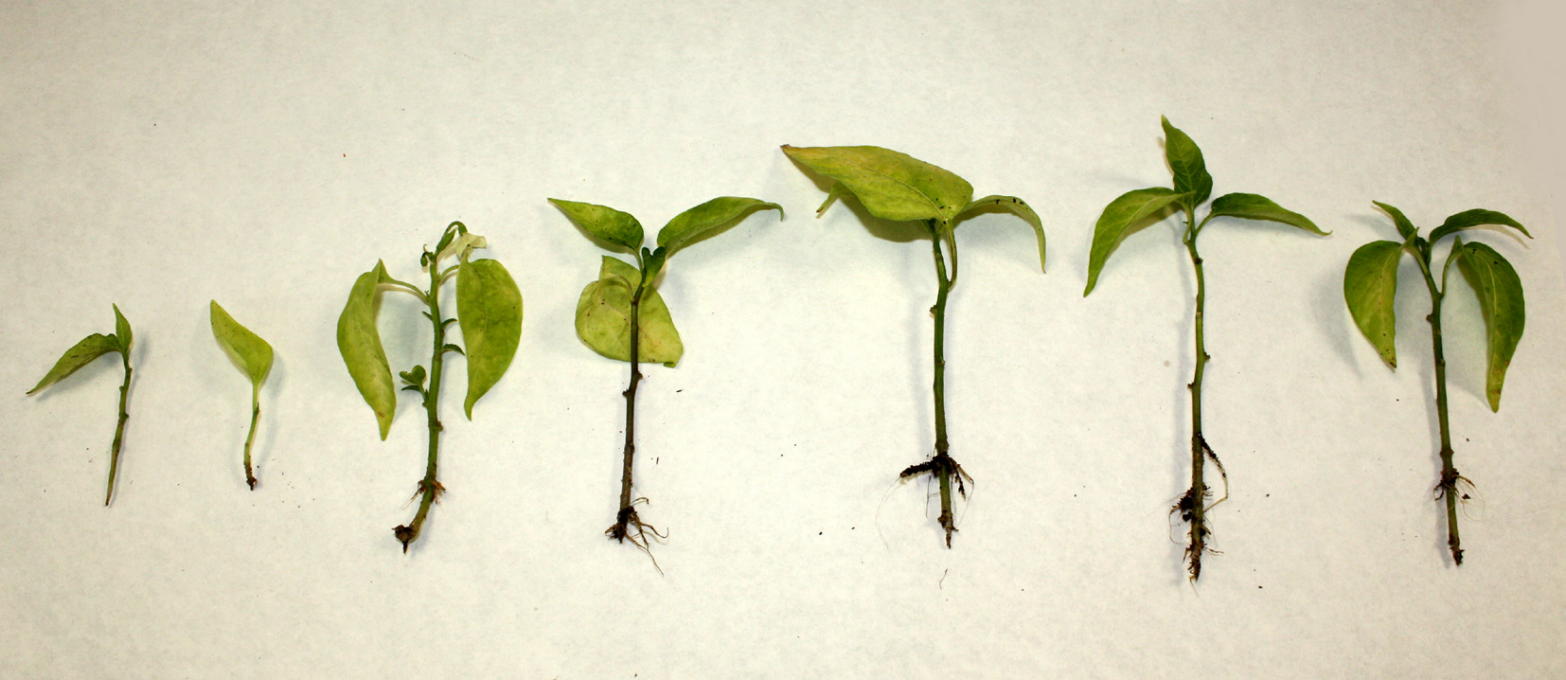
*C*. *frutescens* (♀) × *C*. *annuum* (♂) hybrids showing dwarfism and poor root development at 100 days after germination.

Otherwise, when *C*. *frutescens* was utilized as pistillate parent, only 4 out of 12 possible combinations were successful, in addition, once germinated they showed dwarfism (Table 5 and Fig 5). In this regard, while some authors found partial embryo abortion [41], Yazawa et al. [40] obtained dwarfism in all hybrids obtained and concluded that dwarfism is controlled by two complementary dominant genes. Thus, *C*. *frutescens* (♀) × *C*. *annuum* (♂) crosses seem to follow the Bateson–Dobzhansky–Muller (BDM) model, which explains the simplest scenario for generating genetic incompatibilities without detriment to either of two diverging lineages. The ‘classical’ BDM model involves two epistatic loci aaBB and AAbb wich are innocuous in their native context but interact negatively in hybrids [42].

**Table 5:** Descriptive results of the bridge cross technique using *C*. *frutescens* as genetic bridge between *C*. *annuum* and *C*. *baccatum*. Those F_1_ combinations which did not set fruit, or set fruit but seeds did not germinate, are not included in the table.

Pistillate (♀) accessions	×	Staminate (♂) accessions	No. crosses	No. set fruit (fruit/crosses %)		Germination (%)	Hybrid appearance
Alternative 1: First *C*. *annuum* × *C*. *frutescens* hybrids, followed by hybridization with *C*. *baccatum*							
*C*. *frutescens*	×	*C*. *annuum*					
B144	×	Piq	10	4	(40%)	3/10 (30%)	Dwarfism
B144		Pas	10	8	(80%)	1/10 (10%)	Dwarfism
B144		Bie	10	7	(70%)	1/10 (10%)	Dwarfism
B144		Gui	10	4	(40%)	1/10 (10%)	Dwarfism
Alternative 2: First *C*. *baccatum* × *C*. *frutescens* hybrids, followed by hybridization with *C*. *annuum*							
*C*. *frutescens*	×	*C*. *baccatum*					
B144	×	AjíR	10	2	(20%)	9/10 (90%)	VLS^1^
B144		AjíA	10	1	(10%)	1/10 (10%)	VLS
B144		BrP	10	6	(60%)	3/10 (30%)	VLS

^1^VLS = virus-like sindrome.

Regarding the crossing between *C*. *baccatum* and *C*. *frutescens*, hybrids were only obtained when the latter was utilized as pistillate parent (Fig 4) as was early observed by Rao et al. [43]. However, in agreement with Pickersgill [44] and the results of this experiment with *C*. *chinense*, all hybrids were also affected by virus-like syndrome (VLS), probably due to *C*. *frutescens* cytoplasmic genes, which interact with *C*. *baccatum* nuclear genes. In addition, although Rao et al. [43] were able to recover normal hybrids in appearance, they were sterile. Because of that, despite more *C*. *frutescens* genotypes could be tested for compatibility with *C*. *annuum* or *C*. *baccatum*, in our opinion *C*. *frutescens* should not be used as bridge between both species.

### *In vitro* rescue of immature embryos from crosses between *C*. *annuum* and *C*. *baccatum*

**Fig 6:**
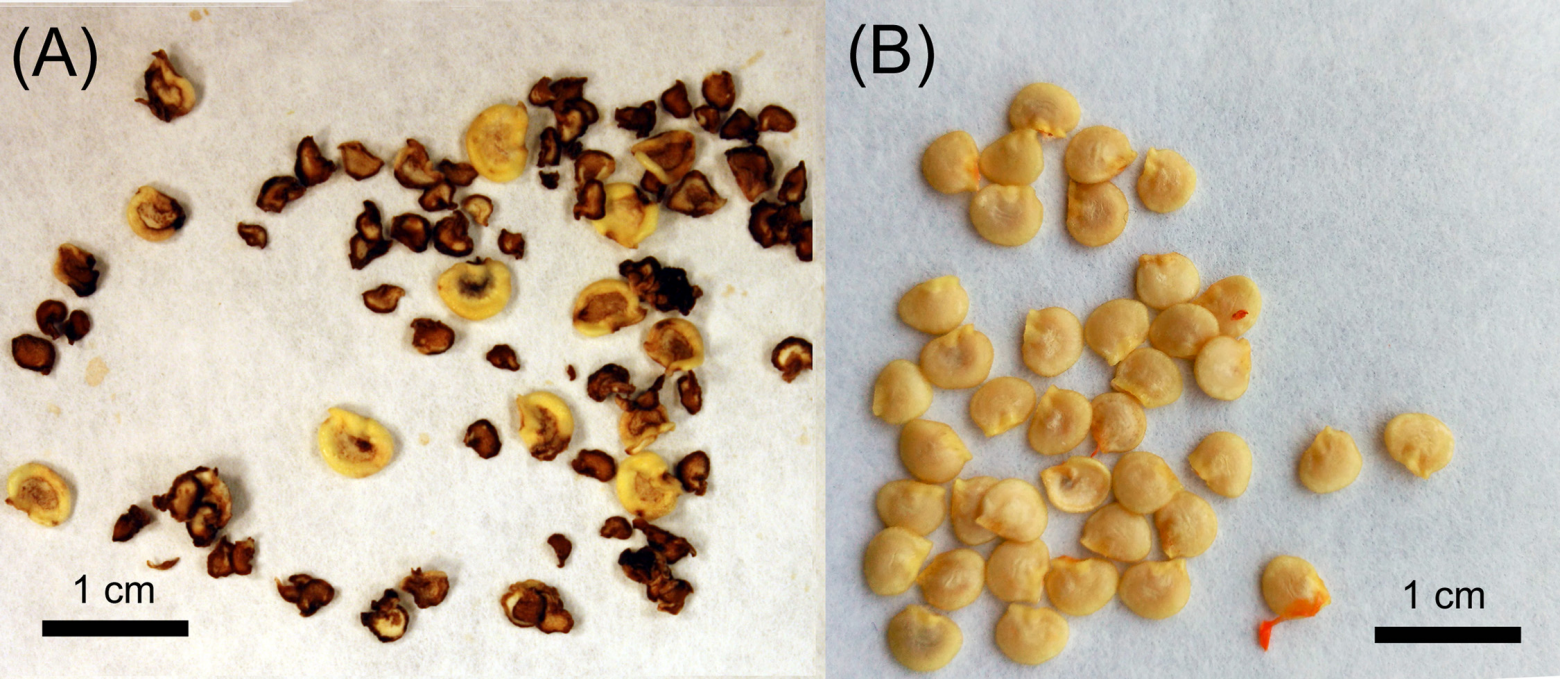
Comparison between normal and aborted seeds from crosses between *C*. *annuum* and *C*. *baccatum*. (A) aborted seeds from interspecific cross between Bierzo (♀) × Aji Rojo (♂), and (B) normal seeds from self-pollinated *C*. *annuum* cv. Bierzo.

**Fig 7:**
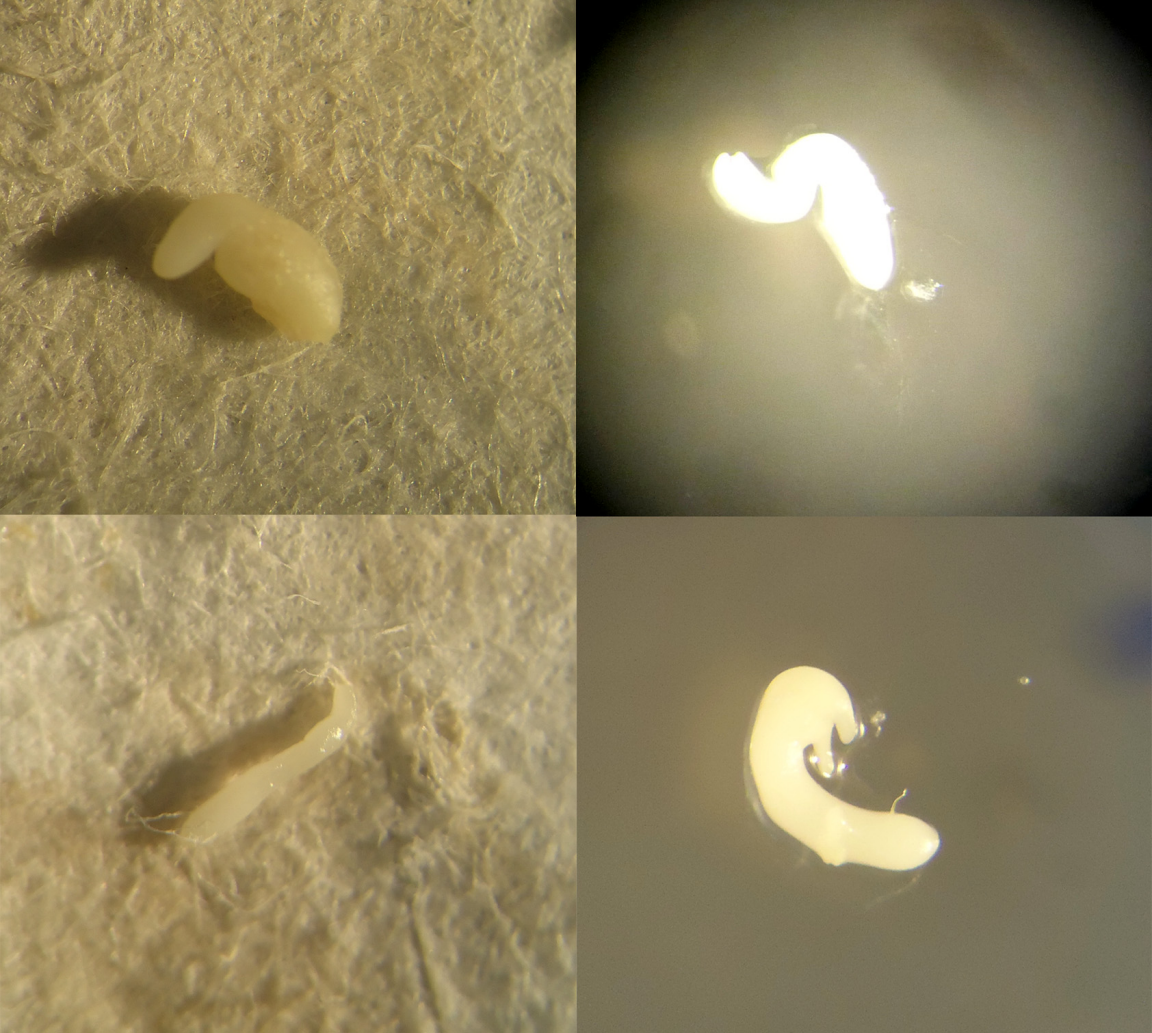
Isolated embryos from interspecific crosses between *C*. *baccatum* and *C*. *annuum* before abortion. Different deformities, due to early hardening of endosperm, can be observed.

In a preliminary experiment, a diallel cross scheme was done between eight accessions of *C*. *annuum* (California Wr. both red and yellow, Arnoia, Bierzo, Guindilla, Pasilla, Piquillo, and Serrano) and the three *C*. *baccatum* accessions. The few set fruits achieved were hasvested at the fully ripe stage and their seeds were removed and evaluated. All seeds were empty and showed necrotic points in the center (Fig 6), which is frequently related to embryo abortion. In fact, as expected, germination tests demonstrated that these seeds were not viable. Those results were in agreement with other authors [6, 23] and confirmed that embryo abortion is an important barrier in crosses between *C*. *annuum* and *C*. *baccatum*. In addition, further anatomical studies of interespecific embryos close to abortion revealed that the main cause of abortion was an early hardening of the endosperm, which limited the development of the embryos and caused morphological deformities and, eventually, embryo collapse and death (Fig 7). Therefore, embryo rescue was the only alternative to achieve these interspecific hybrids.

As a whole, the use of *C*. *baccatum* accessions as male parents provided the highest efficiency in terms of both fruit set and number of regenerated hybrids. Thus, fruit set was recorded in 67% of *C*. *annuum* (♀) × *C*. *baccatum* (♂) combinations (26 out of 36), although the efficiency rate ranged from 4% to 30%, depending on the genotypes involved (Table 6). Interspecific embryos could be excised and cultured *in vitro* from half of these combinations, while no embryos were found in the other half, which might be due to embryo abortion at very early stages (globular or earlier), whose rescue and regeneration would be extremely difficult or unfruitful [29, 36]. Finally, plantlets from 10 *C*. *annuum* (♀) × *C*. *baccatum* (♂) combinations were obtained and reached the adult stage. The efficiency of this *in vitro* technique varied among combinations, ranging from 6% to 75% in P534 × AjíR and P716 × AjíR, respectively (Table 6). Furthermore, combinations involving Ají Rojo and Brazilian Pumpkin *C*. *baccatum* accessions yielded the highest number of *C*. *annuum* (♀) × *C*. *baccatum* (♂) individuals: 36 and 16 hybrid plants, respectively, while only 3 hybrid plants from a single combination were obtained with Ají Amarillo (Table 6).

**Table 6:** Descriptive results of the direct cross and embryo rescue technique to obtain hybrids between *C*. *annuum* and *C*. *baccatum*. F_1_ combinations that did not set fruit are not included in the table.

Pistillate (♀) accessions	×	Staminate (♂) accessions	No. crosses	No. set fruit (fruit/crosses %)		*In vitro* germination (%)	Pollen viability (% ± SD)
*C*. *annuum*	×	*C*. *baccatum*					
Arn	×	AjíA	22	3	(14%)	embryo not available	-
Arn	×	AjíR	50	6	(12%)	embryo not available	-
Arn	×	BrP	45	4	(9%)	embryo not available	-
Bie	×	AjíA	20	3	(15%)	0/5 (0%)	-
Bie	×	AjíR	47	3	(6%)	1/12 (8%)	15 ± 1
Bie	×	BrP	45	6	(13%)	7/14 (50%)	5 ± 4
Bola	×	AjíA	10	2	(20%)	embryo not available	-
Bola	×	AjíR	35	2	(6%)	embryo not available	-
Bola	×	BrP	35	11	(31%)	19/30 (63%)	12 ± 6
CWr	×	AjíA	20	1	(5%)	embryo not available	-
CWr	×	AjíR	45	7	(16%)	0/12 (0%)	-
CWr	×	BrP	45	6	(13%)	embryo not available	-
CWy	×	AjíR	22	2	(9%)	0/15 (0%)	-
Gui	×	AjíA	20	6	(30%)	embryo not available	-
Gui	×	AjíR	48	8	(17%)	5/15 (33%)	14 ± 8
Gui	×	BrP	40	4	(10%)	1/12 (8%)	19 ± 8
Num	×	BrP	25	1	(4%)	embryo not available	-
P534	×	AjíR	35	2	(6%)	1/18 (6%)	10 ± 7
P534	×	BrP	25	1	(4%)	4/18 (22%)	14 ± 4
P716	×	AjíA	10	1	(10%)	3/12 (25%)	0
P716	×	AjíR	35	4	(11%)	9/12 (75%)	20 ± 5
P716	×	BrP	25	2	(8%)	5/14 (35%)	6 ± 2
Pas	×	AjíA	25	1	(4%)	embryo not available	-
Pas	×	AjíR	40	2	(5%)	embryo not available	-
Piq	×	AjíR	40	2	(5%)	embryo not available	-
Ser	×	AjíA	25	1	(4%)	embryo not available	-
*C*. *baccatum*	×	*C*. *annuum*					
AjíR	×	Arn	35	4	(11%)	0/4 (0%)	-
AjíR	×	Bie	35	4	(11%)	1/3 (33%)	33 ± 6
AjíR	×	Bola	10	1	(10%)	embryo not available	-
AjíR	×	Gui	25	3	(12%)	0/10 (0%)	-
AjíR	×	Num	25	1	(4%)	embryo not available	-
AjíR	×	Pas	25	2	(8%)	0/12 (0%)	-
AjíR	×	Piq	25	2	(8%)	2/12 (17%)	21 ± 8
AjíR	×	Ser	25	2	(8%)	embryo not available	-
AjíA	×	Num	10	4	(40%)	embryo not available	-
AjíA	×	Piq	25	1	(4%)	embryo not available	-
BrP	×	Arn	35	1	(3%)	embryo not available	-
BrP	×	Pas	35	1	(3%)	embryo not available	-
BrP	×	Piq	40	1	(3%)	embryo not available	-
BrP	×	Ser	40	2	(5%)	embryo not available	-

On the other hand, a considerably lower efficiency was found in *C*. *baccatum* (♀) × *C*. *annuum* (♂) hybridizations and fruit set fruit was only observed in 14 out of the 36 possible combinations (Table 6). In addition, only three hybrid plants from two combinations, Ají R × Bie and Aji R × Piq, were eventually obtained (Table 6), consequently, breeders are advised to use *C*. *annuum* as female parent in crosses with *C*. *baccatum* to achieve the highest efficiency.

In both cases, pollen fertility was relatively low, comprised between 0% and 33% of P716 (♀) × Aji A (♂) and Ají R (♀) × Bie (♂) (Table 6), which was comparatively lower than the values recorded in the GB strategy. In any case, such values are in agreement with those reported by other authors [24, 45].

Finally, also supported by *in vitro* rescue, BC_1_ generations towards *C*. *annuum* were attempted. Each hybrid was backcrossed with the corresponding *C*. *annuum* parent for compatibility reasons. All BC_1_ crosses involving hybrids as male parents failed and no fruit was obtained. As a result, fruit set was only achieved when hybrids were utilised as female parents. Furthermore, BC_1_ materials were only achieved with *C*. *annuum* (♀) × *C*. *baccatum* (♂) hybrids, while all BC_1_ crosses using *C*. *baccatum* (♀) × *C*. *annuum* (♂) hybrids failed (Table 7). Despite fruit set was low and, consequently, only three BC_1_ combinations were obtained, *in vitro* rescue enabled to regenerate a high number of embryos (Table 7).

**Table 7:** Descriptive results of the backcross and embryo rescue technique to obtain hybrids between *C*. *annuum* (Ca) and *C*. *baccatum* (Cb). BC_1_ combinations that did not set fruit are not included in the table.

Pistillate (♀) accessions	×	Staminate (♂) accessions	No. crosses	No. set fruit (fruit/crosses %)		*In vitro* germination (%)	Pollen viability (% ± SD)
(*Ca* × *Cb*)	×	*C*. *annuum*					
Bie × AjíR	×	Bie	20	0	(0%)	-	-
Bie × BrP	×	Bie	20	2	(10%)	8/16 (50%)	35 ± 4
Bola × BrP	×	Bola	20	4	(20%)	7/16 (44%)	91 ± 4
Gui × AjíR	×	Gui	20	0	(0%)	-	-
Gui × BrP	×	Gui	20	0	(0%)	-	-
P534 × AjíR	×	P534	20	0	(0%)	-	-
P534 × BrP	×	P534	20	0	(0%)	-	-
P716 × AjíA	×	P716	20	0	(0%)	-	-
P716 × AjíR	×	P716	20	0	(0%)	-	-
P716 × BrP	×	P716	20	4	(20%)	8/16 (50%)	40 ± 4
(*Cb* × *Ca*)	×	*C*. *annuum*					
AjíR × Bie	×	Bie	20	0	(0%)	-	-
AjíR × Piq	×	Piq	30	0	(0%)	-	-

Moreover, these BC_1_ materials showed a normal appearance and their pollen viability (35–91%) was considerably higher than the values showed by their corresponding F_1_ parents. Thus, for example Bola (♀) × BrP (♂) had 12% pollen viability, while the corresponding BC_1_ (Bola (♀) × BrP(♂)) (♀) × Bola (♂) had a pollen viability of 91% (Tables 6 and 7). These results demonstrate that pollen fertility in crosses between *C*. *annuum* and *C*. *baccatum* can be recovered fast in 1–2 backcrosses, which may facilitate breeding programs.

## Conclusions

**Fig 8:**
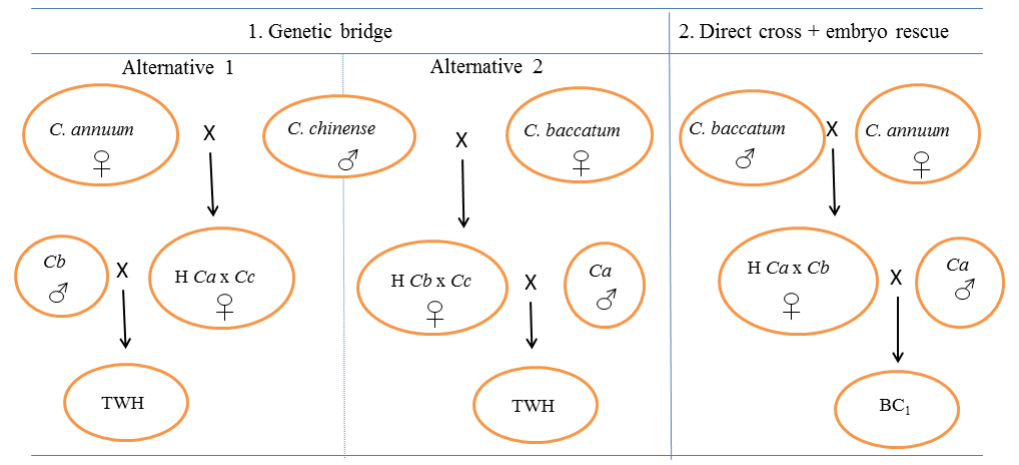
Recommended diagram for wide hybridization between different species for introgression breeding of *C*. *baccatum* to *C*. *annuum* based on the results of this work. Vertical arrows indicate the hybrid obtained, H = Hybrid, Ca = *C*. *annuum*, Cb = *C*. *baccatum*, Cc = *C*. *chinense*, BC_1_ = First Backcross, TWH = Three way hybrid.

According to the results, wide hybridization between *C*. *annuum* and *C*. *baccatum* is possible using both the GB and ER approaches, although the degree of success is highly dependent on the genotype to obtain interspecific hybrids and subsequent generations. The best crossing schemes to obtain successful hybridization and introgression from *C*. *baccatum* to *C*. *annuum* have been identified (Fig 8), and the genotypes with the best performance in these experiments are good candidates for introgression breeding from *C*. *baccatum* to *C*. *annuum*. Ultimately, these results provide breeders with relevant information on wide hybridization approaches and on appropriate plant material to be used for successfully incorporate the *C*. *baccatum* gene pool as a source of variation for introgression breeding in *C*. *annuum* breeding programmes. Additionally, those breeders interested in introgressing genes of interest in *C*. *annuum* from *C*. *chinense* or *C*. *frutescens* should consider the prezygotic and postzygotic barriers between these species identified here in order to achieve a high efficiency.
